# Genome sequencing of *Pseudomonas aeruginosa* phages; UF_RH7 and UF_RH9

**DOI:** 10.1128/mra.01050-23

**Published:** 2024-03-14

**Authors:** Abdolrazagh Hashemi Shahraki, Majid Vahed, Mehdi Mirsaeidi

**Affiliations:** 1 Division of Pulmonary, Critical Care and Sleep, College of Medicine-Jacksonville, University of Florida, Jacksonville, Florida, USA; Loyola University Chicago, Chicago, Illinois, USA

**Keywords:** genome sequencing, *Pseudomonas aeruginosa*, bacteriophages, UF_RH7, UF_RH9

## Abstract

We have sequenced the genomes of two lytic phages, UF_RH7 and UF_RH9, which infect *Pseudomonas aeruginosa*. UF_RH7 belongs to *Casjensviridae* family and has a genome length of 58,217 bp and encodes 82 proteins. UF_RH9 belongs to *Caudoviricetes* class and has a genome length of 42,609 bp and encodes 55 proteins.

## ANNOUNCEMENT

Bacteriophages are abundant in different environments and can be used for elimination of harmful bacteria (1, 2). The collected raw sewage sample from a treatment plant (Alexander Orr Water Treatment Plant located at Miami, Florida, USA) was filtered through 0.2 micron filter and was added to fresh broth culture of *P. aeruginosa* (strain DJ06) (10 µL sewage was mixed with 400 µL bacteria). The mixture was incubated at 37°C (20 min) and was cultured using double-layer agar by incubating the plates at 37°C (24 h) (3). Two different phages were isolated. The phages were purified via single plaque isolation and then purified after five rounds of single plaque isolation. DNA was extracted from 5 mL lysate of each phage (QIAamp MinElute Virus Kit, Qiagen, USA). DNA library for phages were prepared using Illumina’s Nextera XT library preparation kit and were sequenced using Illumina NovaSeq 6000 (paired-end 150 cycle). Bcl2fastq v2.20 (Illumina) was used to de-multiplex reads. Cutadapt program v2.8 was utilized to remove sequencing adaptors and low-quality bases (4). *P. aeruginosa* DNA was removed from the data using the read mapper of the STAR package v.2.5 (5). MetaWRAP v1.2.00 (6) was used to assemble the unmapped paired-end reads using NCBI Viral databases. QUAST v5.0.2 (7) was used to measure the quality analysis of the data. Centrifuge v1.04b (8) was used to analyze the assembled consensus sequences (>15,000 bp). Each phage had only one assembled contig over 15,000 bp. Consend v.29 was used to determine to phage termini (9). CheckV v1.0.1 was used to determine the completeness and accuracy of phage genome (10). Taxonomic identity of the sequences was determined by NCBI BLASTn [Nucleotide collection (nr/nt)] (11). Open reading frames (ORFs) were defined for each virus by GeneMarkS v2 (12), and the genome of each phage was annotated by PHASTER and BLASTp (non-redundant protein sequence) (11, 13). The presence of tRNA sequences, virulence factors, antibiotic resistance genes were evaluated by tRNAscan-SE v2.0 (14), ResFinder v4.0 (15), and Antibiotic Resistance Genes Database (16), respectively. Default parameters were used for software tools.

After post trimming and filtration of bacterial host sequences, the genome was assembled for both phages. The *Pseudomonas* phage UF_RH7 genome is a 58,217 bp long with circular double-stranded DNA (dsDNA) genome with a GC content of 56.4%, 21,196,642 total reads, and 29,432 × average read coverage. The *Pseudomonas* phage UF_RH9 genome is a 42,609 bp long with linear dsDNA genome with a GC content of 53.6%, 53,009,336 total reads, and 21,391 × average read coverage.

CheckV results showed high-quality (>90% completeness) and high confidence (0%–5% error) of the genome of each phage. UF_RH7 and UF_RH9 include 82 and 55 ORFs, respectively. UF_RH7 only shares high DNA similarity (~81%) and 70% sequence coverage with *Pectobacterium* phage MA11 (GenBank: NC_053014) and *Pectobacterium* phage MA12 (GenBank: NC_053015). UF_RH9 shares high DNA similarity to our previously reported *Pseudomonas* phages UF_RH1 (17) and UF_RH5 (18) (Table 1). In the genomes of both phages, we did not detect any tRNA sequences, virulence genes, or antibiotic resistance genes.

**TABLE 1:** Genome sequence coverage (top number in each cell) and nucleotide identity (bottom number) of UF_RH9 with their closest relatives

Phages (GenBank accession number)	Percentage of sequence coverage and similarity						
	UF_RH9	UF_RH1 (NC_072810.1)	UF-RH5 (OQ319036.1)	vB Pae-Kakheti25 (JQ307387.1)	PA02_HSun-2022 (OM234791.1)	Epa41 (MT118305.1)	Epa40 (MT118304.1)
UF_RH9	100 100	99 99	99 100	95 96.2	95 97.2	94.8 92	94.8 91
UF_RH1 (NC_072810.1)	99 99	100 100	99.9 99	96.2 95	97.2 95	97 97.9	94.8 92
UF-RH5 (OQ319036.1)	99 100	99.9 99	100 100	96.8 95	97.2 95	95 97	97.2 92
vB Pae-Kakheti25 (JQ307387.1)	95 96.2	96.2 95	96.8 95	100 100	98 98	96.8 91	96.8 90
PA02_HSun-2022 (OM234791.1)	95 97.2	97.2 95	97.2 95	98 98	100 100	97.2 92	97.2 91
Epa41 (MT118305.1)	94.8 92	94.8 92	97.2 92	96.8 91	97.2 92	100 100	100 99.9
Epa40 (MT118304.1)	94.8 91	94.8 91	97.2 91	96.8 90	97.2 91	100 99.9	100 100

## Data Availability

The complete phage genome sequence was deposited in GenBank under the accession numbers OR589135 and OR604635 for UF_RH7 and UF_RH9, respectively. The raw data for UF_RH7 are available in the NCBI Sequence Read Archive (SRA) under BioProject accession number PRJNA1019333, SRA accession number SRR26125953, and BioSample accession number SAMN37480981. The raw data for UF_RH9 are available in the NCBI Sequence Read Archive (SRA) under BioProject accession number PRJNA1021600, SRA accession number SRR26223087, and BioSample accession number SAMN37563411.
